# The implications of robot navigation in social space: perceptual effects of socially aware and baseline navigation

**DOI:** 10.3389/frobt.2026.1833485

**Published:** 2026-06-12

**Authors:** Kristina Nikolovska, Francesco Maurelli, Arvid Kappas

**Affiliations:** 1 School of Computer Science and Engineering, Constructor University, Bremen, Germany; 2 School of Business, Social and Decision Sciences, Constructor University, Bremen, Germany

**Keywords:** human perception, human-robot interaction, robot navigation strategy, shared environments, social navigation, socially aware navigation, user evaluation

## Abstract

**Introduction:**

Robot navigation in shared environments may influence how robots are socially interpreted by nearby users, beyond its purely functional role in task execution. Prior work has shown that individual navigation features, such as proximity regulation, approach direction, or speed adaptation, can affect user perception; however, fewer studies have examined how these behaviors operate when combined within a single socially aware navigation framework. Understanding how integrated navigation behaviors shape user perception is important for designing robots that can operate appropriately and comfortably in human-centered environments.

**Methods:**

We conducted a perceptual evaluation of a socially aware navigation framework that integrates five socially relevant navigation behaviors, including approach direction constraints, side-consistent passing behavior, proximity-based speed adaptation, and user-oriented motion cues. A controlled in-person user study was conducted with 40 participants comparing this socially aware navigation strategy against a baseline costmap-based obstacle avoidance strategy during repeated navigation encounters with a Pepper robot in an office-like environment.

**Results:**

The socially aware navigation strategy was associated with more positive evaluations across multiple social and movement-related dimensions, including warmth, likeability, animacy, perceived safety, naturalness of motion, and overall movement preference, while maintaining comparable ratings of competence and comfort. Open-ended responses further indicated that participants frequently described navigation behavior in experiential and social terms related to comfort, awareness, friendliness, and attentiveness.

**Discussion:**

These findings suggest that combined socially informed navigation behaviors can meaningfully influence how robot motion is perceived during human–robot interaction. More broadly, the results highlight the importance of designing navigation systems that are not only collision-free and efficient, but also socially interpretable and considerate of nearby users in shared environments.

## 1 Introduction

Robot motion in shared environments is not perceived as neutral. We have previously shown that even very simple aspects of robot motion are interpreted in terms of agency and related concepts that attribute a form of self to the machine Pohl et al. (2026), Pohl et al. (2025), Nikolovska et al. (2024). Long before task success or system performance can be evaluated, navigation behavior may shape how robots are interpreted and socially evaluated by human observers Guillén-Ruiz et al. (2023), Scales et al. (2024), Mavrogiannis et al. (2022). In these everyday shared spaces, it is typically lay users, bystanders, or interaction partners who evaluate whether a robot’s motion feels appropriate, trustworthy, or disruptive. In such contexts, how a robot moves - how it approaches, passes, adapts its speed, and positions itself relative to people -functions as a communicative signal that may convey awareness, intent, and social sensitivity Francis et al. (2023), Karwowski et al. (2024), Shiba et al. (2025). Understanding what constitutes socially acceptable robot navigation has therefore become an important topic in human–robot interaction research, and one that increasingly needs to be addressed as robots become integrated into public and semi-public spaces Singamaneni et al. (2024), Karwowski et al. (2024).

This perspective has driven the emergence of socially aware navigation (SAN), an approach that integrates social cues into motion planning to make robots appear more natural and considerate in shared spaces Guillén-Ruiz et al. (2023), Möller et al. (2021). However, existing research tends to examine navigation behaviors in focused scenarios or with a limited set of parameters. For example, Neggers et al. (2022) investigated how robot speed influences comfortable passing distances; Shiba et al. (2025) examined how extending the robot’s reaction distance beyond typical interaction ranges affects perceived safety, smoothness, and politeness; and Mavrogiannis et al. (2022) focused on approach direction in crowded environments. Comparatively fewer studies have examined navigation strategies that combine multiple socially relevant navigation adaptations within a single behavioral configuration Karwowski et al. (2024), Francis et al. (2023), despite growing calls for broader perception-oriented evaluation frameworks in social navigation Francis et al. (2023), Singamaneni et al. (2024).

The present study adopts a user-centered evaluation perspective grounded in empirical insights into how people expect robots to move in shared environments. The socially aware navigation strategy evaluated in this study was developed based on findings from our previous work, which showed that users consistently prioritize aspects such as approach direction, passing behavior, speed adaptation, and spatial positioning when evaluating the social appropriateness of robot navigation in hypothetical shared-space scenarios Nikolovska et al. (2025). In that work, participants rated their preferences for different navigation behaviors described through text-based scenarios without direct exposure to a physical robot. Building on these findings, the identified user expectations were translated into a set of modular social navigation layers and integrated into a unified navigation framework Nikolovska et al. (2026a). While this prior work established a principled link between user expectations and navigation system design, it did not examine whether such design choices produce perceptually meaningful differences during real human–robot interaction.

The present study addresses this gap by investigating whether an integrated socially aware navigation strategy, combining multiple social navigation principles within a single navigation framework, produces differences in users’ perceptual evaluations during a real in-person encounter with a mobile robot. By connecting user expectations, navigation system design, and perceptual evaluation, this work contributes a multi-stage investigation of socially aware navigation spanning expectation elicitation, behavioral implementation, and human perception. To this end, we report a controlled in-person experiment in which participants shared space with a mobile robot following different navigation strategies in an office-like indoor environment, allowing us to examine how robot motion influences social evaluations during real interaction encounters.

Based on prior findings in social navigation and human–robot interaction, we formulate the following hypotheses: H1a (Perceived social quality). A robot employing the proposed socially aware navigation strategy-combining approach direction constraints, side-consistent passing behavior, proximity-based speed modulation, and nonverbal awareness signaling-is expected to receive higher ratings on positive social and movement-related attributes, including warmth, likeability, animacy, naturalness and smoothness of motion, as well as overall movement preference, compared to the same robot using a baseline costmap-based obstacle avoidance strategy. This prediction is grounded in prior findings showing that individual navigation features, such as approach direction, proxemic distance, and speed modulation, influence social evaluations of robot behavior Okunevich et al. (2023), Sorrentino et al. (2021), Mavrogiannis et al. (2019), and extends these findings to a combined multi-component navigation strategy derived from empirically elicited user expectations Nikolovska et al. (2025). H1b (Comfort). A robot employing the proposed socially aware navigation strategy will be rated as more comfortable than the same robot using a baseline naive costmap-based obstacle avoidance strategy, based on the expectation that increased interpersonal distance and socially consistent path choices reduce perceived discomfort during shared-space navigation. H2 (Overall preference). A majority of participants will indicate an overall preference for the socially aware navigation strategy over the baseline navigation strategy when asked to choose between the two.

The remainder of this paper is organized as follows: Section 2 reviews related work on socially aware navigation, Section 3 describes the navigation strategies and experimental design, Section 4 presents the study results, and Sections 5 and 6 discuss the findings and draw conclusions.

## 2 Background

### 2.1 Navigation motion as social communication in human–robot interaction

Humans automatically and often unconsciously interpret motion as socially meaningful, inferring intentions and social cues from movement and approach behavior Wen and Imamizu (2022). In human interaction, non-verbal communication plays a central role in conveying intentions, attitudes, and social norms, with meaning routinely inferred from body movement, timing, spatial positioning, and approach behavior Cross and Kappas (2026). This sensitivity extends to artificial agents: people automatically and unconsciously interpret robot behavior as socially meaningful, even when a robot is not explicitly designed to be social Spatola and Wudarczyk (2021), Ju and Takayama (2009), Pohl et al. (2025), Nikolovska et al. (2024). As a result, robot motion does not merely serve functional goals such as reaching a destination or avoiding obstacles; rather, movement itself functions as a powerful non-verbal social stimulus that appears to indicate intentions, affective states, and social stance, shaping how robots are perceived and evaluated Hostettler et al. (2023), Francis et al. (2023). While non-verbal communication in HRI has traditionally been associated with expressive modalities such as gaze, gestures, or facial cues Tatarian et al. (2022), Kovács et al. (2025), Saunderson and Nejat (2019), movement itself, and in particular navigation motion, plays an important role in how social meaning is inferred from robot behavior Mead and Mataric (2017), Daza et al. (2021).

Unlike discrete expressive signals, navigation is continuous, persistent, and unavoidable in shared environments. Every navigation decision, including where a robot moves, how fast it travels, and how it positions itself relative to people, is immediately observable and open to social interpretation Rios-Martinez et al. (2015), Francis et al. (2023), Pohl et al. (2025). Consequently, navigation motion often shapes first impressions and ongoing judgments, even in the absence of explicit interaction Mavrogiannis et al. (2022). A range of navigation-related motion features have been shown to shape how people interpret robot behavior. Variations in movement speed, acceleration, and pauses influence perceived urgency, confidence, and politeness, while motion timing can implicitly entrain human behavior and expectations Karwowski et al. (2024), Che et al. (2020). Through these temporal properties alone, robots seem to express attentiveness or disregard, even in the absence of explicit communication Che et al. (2020), Mavrogiannis et al. (2022).

Trajectory shape and motion legibility further contribute to how navigation behavior is socially interpreted Singamaneni et al. (2024), Nikolovska et al. (2024). In navigation contexts, legible motion, such as clearly structured trajectories or motion-coupled cues indicating intended direction, supports observers in inferring a robot’s goals and future actions. The absence of such legibility has been associated with reduced social acceptability, whereas transparent and predictable motion increases comfort and trust in shared environments Hetherington et al. (2021), Francis et al. (2023), Mavrogiannis et al. (2022). In this sense, navigation motion becomes not only functionally safe but also socially transparent.

Proxemics and approach behavior constitute another critical dimension of how navigation motion is socially interpreted. A robot’s approach distance, path selection, and orientation relative to a person significantly influence perceived social presence and attributed emotional states in encounters such as hallways or shared corridors. Prior work has shown that these motion-based cues can outweigh isolated expressive signals, such as gaze alone, in shaping social judgments Rios-Martinez et al. (2015), Mead and Mataric (2017). Similarly, approach speed and movement style act as cues for urgency, dominance, or courtesy, positioning navigation behavior as a primary medium through which intentions are inferred Truong and Ngo (2018), Truong and Ngo (2017), Gil et al. (2021).

Social meaning can also emerge from navigation motion in the absence of explicit interaction or task relevance. Navigation behaviors that implicitly express attention, sensitivity, or responsiveness through motion alone have been shown to significantly alter human perception of robots, highlighting movement as a sufficient carrier of social information Nikolovska et al. (2024), Che et al. (2020).

Together, these findings underscore that navigation motion is not socially neutral: it is continuously interpreted and evaluated by human observers as socially meaningful. If navigation motion is perceived as a social signal, its evaluation cannot be reduced to isolated parameters or individual motion cues. Human observers experience navigation as a continuous, integrated behavior unfolding over time, yet much of the existing literature evaluates navigation features in isolation. In real-world settings, users encounter robots without prior knowledge of their underlying navigation algorithms or design principles. Social evaluations therefore emerge directly from observable motion behavior, making it important to understand how integrated navigation strategies are perceived during real encounters. Whether users can meaningfully distinguish and evaluate complete navigation strategies as coherent social behaviors therefore remains a critical open question.

### 2.2 Socially aware navigation: from parameters to principles

Early mobile robot navigation systems were primarily designed around geometric and kinematic objectives such as collision avoidance, shortest paths, and dynamic feasibility Katona et al. (2024), Liu et al. (2023), Sánchez-Ibáñez et al. (2021). In these frameworks, humans are typically modeled as moving obstacles whose trajectories must be predicted and avoided Shiomi et al. (2014). While such approaches can be technically safe, they often yield behavior that is socially inappropriate or uncomfortable for nearby people, for example by cutting too close, blocking passages, or moving in ways that feel abrupt or inattentive Shiomi et al. (2014).

Initial attempts to improve social acceptability largely focused on tuning low-level parameters rather than explicitly modeling social principles Truong and Ngo (2017). Designers adjusted speed limits near humans, expanded safety margins, or introduced conservative stopping rules in the presence of people Truong and Ngo (2017), Kivrak et al. (2021). Although these heuristics sometimes improved comfort, they were difficult to generalize across contexts and did not provide a principled account of which aspects of navigation mattered most to users, or why Francis et al. (2023).

Subsequent work began to incorporate concepts from human proxemics, crowd dynamics, and social psychology into navigation algorithms, introducing notions such as personal space Rios-Martinez et al. (2015), group formations Truong and Ngo (2017), Shiomi et al. (2014), or socially compliant passing behavior Chen et al. (2019). However, a central challenge lies in formalizing abstract social norms, such as socially appropriate motion, predictability, passing behavior, movement smoothness, and respect for personal space, into computational objectives and constraints for robot navigation Francis et al. (2023). Social navigation behaviors rarely operate in isolation: changes in speed, distance, trajectory shape, and yielding behavior interact in complex ways, and their combined effect is ultimately judged holistically by human observers Francis et al. (2023), Zhou et al. (2024). Despite substantial progress, socially aware navigation therefore remains challenged by how multiple social principles interact and how those interactions are perceived in real environments Francis et al. (2023).

Much of the existing work evaluates navigation components after they have been implemented, concentrating on user reactions to predefined behaviors Zhou et al. (2024). By contrast, understanding socially aware navigation also requires insight into users’ expectations before system design - what people believe a robot *should* do in typical shared - space encounters and which trade-offs they are willing to accept between efficiency and social comfort.

To address this gap, prior work introduced the Preference for Robot Motion (PRoMo) questionnaire, a user-centered instrument designed to capture how people evaluate and prioritize navigation-related behaviors in shared environments through hypothetical text-based navigation scenarios Nikolovska et al. (2025). By eliciting preferences across key navigation dimensions - including safety, predictability, proximity, speed, and responsiveness - PRoMo provides an empirical basis for identifying which navigation principles users consider socially appropriate and how these principles should be balanced when they come into conflict. Importantly, the PRoMo results did not merely describe abstract preferences, but directly informed the design of the socially aware navigation behaviors evaluated in the present study Nikolovska et al. (2026a). The navigation layers used here were derived from these empirically elicited user expectations, translating preference-level insights into concrete planning constraints and motion adaptations. The present work therefore examines the perceptual consequences of embedding user-derived navigation principles into an integrated navigation strategy, linking expectation elicitation, behavior design, and user evaluation.

### 2.3 Perception of integrated navigation behavior

Prior work has identified numerous navigation features that influence how robots are socially evaluated by human observers, including approach direction, passing behavior, interpersonal distance, legibility, and speed adaptation Francis et al. (2023), Gao and Huang (2022), Karwowski et al. (2024). However, many existing studies examine these navigation features individually or within focused interaction scenarios, such as passing or approaching behaviors Gao and Huang (2022), Singamaneni et al. (2024). While such approaches provide valuable insight into specific navigation factors, comparatively fewer studies have evaluated navigation strategies that combine multiple socially relevant navigation adaptations within a single behavioral configuration Francis et al. (2023), Karwowski et al. (2024).

As robots operate in increasingly complex shared environments, users are likely to encounter navigation behavior composed of multiple simultaneously active motion adaptations rather than isolated navigation features presented independently. This motivates the need to examine how broader socially aware navigation configurations are perceived during real interaction encounters. In the present work, we therefore compare complete navigation strategies that differ in their incorporation of socially informed navigation principles, rather than experimentally manipulating individual navigation parameters in isolation.

By focusing on users’ perception of the robot during shared-space interaction, the present study examines whether a socially aware navigation strategy combining multiple navigation adaptations produces perceptually meaningful differences in social evaluations compared to a baseline navigation configuration.

## 3 Methodology

This study investigates how different robot navigation strategies influence users’ perception of a robot in shared environments. To this end, we conducted a controlled in-person user study in which participants observed a mobile robot navigating under two conditions: a baseline navigation strategy and a socially aware navigation strategy. The following sections describe the robot platform and experimental environment, the implemented navigation strategies, the experimental design, the participant sample, and the evaluation measures.

### 3.1 Robot platform and environment

The experiment was conducted using Pepper (Aldebaran Robotics), a humanoid social robot equipped with a holonomic base that enables smooth omnidirectional motion. Pepper was used as a representative social robot platform for studying navigation behavior in shared human–robot environments.

The experiment was conducted in a controlled indoor environment representative of a shared workspace. The environment and user position were kept constant across all trials. For each navigation condition, the robot was assigned the same start and goal poses. As a result, differences in the generated paths reflect the influence of the navigation strategy rather than variations in the navigation task or environmental layout.

**FIGURE 1:**
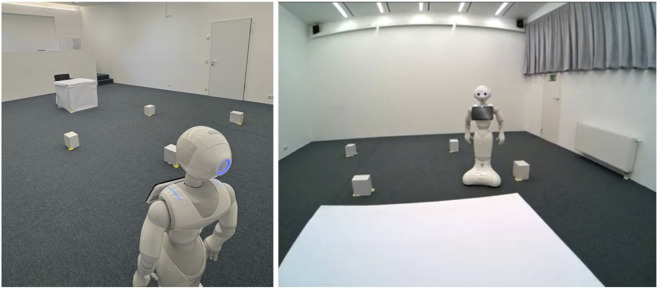
Experimental environment and participant viewpoint. Left: Overview of the experimental space showing the static obstacle layout and the seated user location used during navigation trials. Right: View from the seated user’s position, illustrating how the robot’s navigation behavior was observed.

Participants were seated throughout the experiment to reflect a typical office scenario, in which a person is stationary at a desk while a robot navigates the shared workspace. This design choice was motivated by ecological validity, as seated users represent a common interaction context in office-like environments, and by experimental control, as the fixed position ensured a consistent viewpoint and interaction distance across all participants and trials. Figure 1 shows the experimental environment. The left panel depicts the environment with the seated user’s location, while the right panel shows the environment from the user’s point of view.

### 3.2 Navigation strategies

Two navigation strategies were evaluated: a baseline navigation strategy and a socially aware navigation strategy. Both strategies were implemented within the ROS move_base framework, using GlobalPlanner for global path planning and DWAPlannerROS for local trajectory generation. Localization was performed using the Adaptive Monte Carlo Localization (AMCL) algorithm. This planner configuration was selected because it represents one of the most widely adopted navigation architectures for mobile robots in ROS, offering more flexible global path generation and smoother local obstacle avoidance than alternatives such as navfn and base_local_planner. The configuration was additionally validated through preliminary deployment tests on the Pepper platform. The baseline planner was initialized using standard ROS parameters and subsequently adapted to the robot and environment characteristics. The global planner was configured to use Dijkstra’s algorithm with quadratic approximation and gradient-based path extraction, with an inflation radius of 0.45 m . The local planner was configured according to Pepper’s kinematic constraints, including a maximum linear velocity of 0.55 m/s and a maximum angular velocity of 1.0 rad/s . Goal tolerances were set to 0.15 m for positional accuracy and 0.1 rad for orientation accuracy, while the forward simulation horizon was set to 2.0 s . These planner and localization parameters were applied identically across both experimental conditions. Consequently, the two navigation strategies differed only in whether the additional human-informed navigation layers were active.

The baseline navigation strategy relied solely on the standard ROS costmap configuration, consisting of static, obstacle, and inflation layers. In this condition, the robot generated trajectories based only on free space and obstacle avoidance, treating the seated participant equivalently to any other obstacle in the environment. The resulting behavior, therefore, reflected a primarily task-oriented navigation strategy optimized for geometrically efficient and collision-free path execution. This condition is hereafter referred to as the baseline condition (BL).

The socially aware navigation strategy (SA) extended the baseline configuration through the addition of five modular navigation layers, each encoding a distinct socially informed navigation constraint derived from empirical findings on user navigation preferences Nikolovska et al. (2025). The first component, the *Proximity Layer*, introduced a circular Gaussian cost distribution centered on the detected user position, discouraging close-range trajectories and increasing interpersonal distance without introducing hard exclusion zones. The second component, the *Approaching Layer*, constrained close-range approach trajectories to remain within the user’s forward-facing field of view, defined as a configurable angular sector of approximately 120° , while penalizing approaches from outside this region. The third component, the *Passing Layer*, introduced an asymmetric cost bias that encouraged right-hand passing relative to the robot’s direction of motion, thereby promoting more socially predictable path selection. These three layers modified the global planning costmap and produced systematic deviations from the baseline trajectories. In addition to these path-level adaptations, two motion-level modules were implemented. The *Speed Adjustment Module* dynamically scaled the robot’s commanded velocity according to the user’s position, defining a slowdown region with an approximate radius of 1.2 m together with a smaller frontal stop zone within the user’s field of view, where the robot briefly halted. Finally, the *Head Movement Module* oriented the robot’s head toward the user when entering the frontal stop zone, providing a nonverbal motion cue indicating awareness during close-range encounters. A detailed technical description of all navigation layers, parameter settings, and validation procedures is provided in the companion paper Nikolovska et al. (2026a).

**FIGURE 2:**
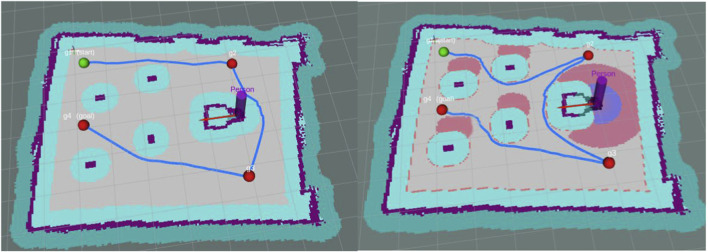
Comparison of baseline and socially aware navigation costmaps and resulting trajectories. Left: Baseline navigation configuration without the socially aware layers. Right: Combined socially aware navigation configuration including the proximity, approaching, and right-side passing layers overlaid on the environment map. The visualization illustrates the resulting spatial cost distributions around the seated user and obstacles, as well as the corresponding trajectory adaptations between identical start and goal positions. Although the right-side passing layer is applied to both static obstacles and the person, its visual effect around the person is partially overridden by the proximity and approaching layers, which dominate the combined social cost distribution in human-centered regions. Speed adaptation is not visualized.

Figure 2 illustrates the socially aware navigation framework, including the user-centered navigation layers and the resulting trajectory differences compared to the baseline condition. The left panel shows the baseline navigation costmap without the socially aware layers, while the right panel shows the combined socially aware configuration. Although the right-side passing layer is applied to both static obstacles and the person, its visual effect around the person is partially overridden by the proximity and approaching layers, which dominate the combined social cost distribution in human-centered regions.

### 3.3 Experimental design

Participants took part in an in-person study conducted individually. Upon arrival, they provided informed consent and completed a short demographic questionnaire. Participants were given a general explanation of the procedure but were not informed about the existence of different navigation strategies. Instead, they were told that the robot would repeatedly navigate the environment as part of an environmental assessment task.

Each participant observed the robot navigating the environment under two conditions: a baseline navigation strategy and a socially aware navigation strategy. In total, participants observed six navigation runs, with three runs per condition. The robot started from three predefined locations, with each run ending at the next starting position, ensuring comparable path lengths and environmental context across runs. The order of navigation conditions was counterbalanced across participants. Within each run, the robot followed a fixed trajectory structure consisting of two predefined intermediate waypoints, ensuring an identical route structure across conditions. The trajectories covered all major areas of the room, including passages close to the seated participant and navigation around static objects.

After each navigation run, participants completed a questionnaire assessing their perception of the robot’s navigation behavior (Section 3.5). After completing all runs, participants were debriefed about the purpose of the study and the differences between the navigation strategies. Throughout the experiment, the experimenter remained in the same room but out of the participant’s line of sight, positioned behind a partition, and monitored the robot remotely to ensure correct execution of the navigation runs.

### 3.4 Participants

A total of *45* participants were recruited for the study. Data from *5* participants were excluded due to incomplete questionnaire responses or invalid navigation trials, defined as runs in which the robot failed to execute the intended navigation behavior. The final sample therefore consisted of *40* participants (*18* female, *21* male, *1* non-binary; M=24.2 , SD=5.4 , range: 18–37 years). Participants were recruited from the university community and participated voluntarily. All participants reported normal or corrected-to-normal vision. No prior experience with robotics was required. The study was reviewed and approved by the Ethics Committee of Constructor University, and all participants provided written informed consent before participation.

### 3.5 Evaluation measures

Participants evaluated the robot’s navigation behavior using a combination of standardized and custom questionnaires. The questionnaires were administered immediately after each individual navigation run, resulting in six separate questionnaire completions per participant in total. Thus, after every navigation interaction, participants completed the full evaluation set again based on the behavior observed during that specific run.

After each run, participants completed a short-form version of the Robot Social Attributes Scale (RoSAS-SF), which assesses perceived *warmth*, *competence*, and *discomfort* using adjective-based ratings Neuenswander et al. (2025). Participants also completed a 10-item form derived from the Godspeed Questionnaire, covering anthropomorphism, animacy, likeability, perceived intelligence, and perceived safety using bipolar adjective pairs (e.g., fake-natural, machinelike-human-like, stagnant-lively, dangerous-safe) Bartneck et al. (2009). The short form was constructed by selecting the highest-loading item pairs for each dimension in order to reduce participant burden while retaining coverage of the five original Godspeed factors. To capture overall movement preference and perceived motion quality, participants additionally rated two custom items after each run: (1) *“I would want the robot to move like this.”* and (2) *“The robot’s movement felt natural and smooth.”* These items were rated using a continuous 0–100 slider scale.

At the end of the experiment, participants completed a post-session questionnaire assessing prior experience with robots, including whether they had previously built, controlled, or interacted with robots, as well as a self-rated assessment of their robotics knowledge on a 5-point scale Schaefer (2016). Finally, participants answered three open-ended questions: *“What differences did you notice in the robot’s movements?”*, *“Which movement style did you prefer, and why?”*, and *“Any suggestions for improving the robot’s movement or behavior?”* All questionnaires were administered digitally using a tablet provided by the experimenter.

### 3.6 Data analysis

Data were analyzed using paired-samples t-tests to compare participant ratings between the Baseline and Social navigation strategies. This approach was selected due to the within-subject design, in which each participant evaluated both navigation conditions. Additional exploratory analyses examined whether participant characteristics moderated the observed effects. Gender and field of study (technical vs. non-technical) were analyzed using independent-samples t-tests on condition difference scores (Social–Baseline). Self-reported robot knowledge (5-point scale) and hands-on robot experience (3-point scale) were examined using Pearson correlations with the same difference scores. No correction for multiple comparisons was applied, as these analyses were exploratory and intended to contextualize the primary results. Open-ended responses were analyzed using a lightweight thematic grouping approach. Recurring keywords and short phrases related to navigation behavior, such as approach direction, interpersonal proximity, head orientation, and motion smoothness, were identified and grouped to support interpretation of the quantitative findings.

## 4 Results

This section reports the effects of navigation strategy on participants’ perceptions of the robot’s motion, based on within-subject comparisons between the Socially Aware and Baseline navigation strategies.

### 4.1 Effects of navigation strategy on perceived robot motion

To examine whether observers perceived differences between the Socially Aware (SA) and Baseline (BL) navigation strategies, paired-samples t-tests were conducted for each questionnaire dimension. Table 1 summarizes descriptive statistics, test results, and effect sizes for all measures. Across multiple measures, the SA navigation strategy received significantly higher ratings than the BL navigation strategy. In particular, participants perceived SA navigation as significantly more natural and smooth $\left(d_{z}=0.68\right)$ and expressed a significantly stronger preference for the robot’s movement behavior under this condition. These preference ratings indicate a clear advantage for socially aware navigation and provide indirect support for H2.

**TABLE 1:** Comparison of participant ratings between Socially Aware (SA) and Baseline (BL) navigation strategies. Values are reported as mean ± standard deviation. Positive mean differences indicate higher ratings for SA navigation.

Measure	N	SA mean	BL mean	Mean Diff.	t	p	$d_{z}$
Naturalness/Smoothness	40	55.78 ± 18.63	45.07 ± 17.95	10.72	4.28	< 0.001	0.68
Animacy	40	4.05 ± 1.19	3.49 ± 1.01	0.56	3.56	0.001	0.56
Preference for movement	40	56.41 ± 16.77	47.34 ± 18.91	9.07	3.34	0.002	0.53
Warmth	40	3.69 ± 1.02	3.16 ± 1.26	0.53	3.19	0.003	0.50
Anthropomorphism	40	3.75 ± 1.17	3.34 ± 0.80	0.41	3.02	0.004	0.48
Likeability	40	4.92 ± 0.86	4.55 ± 1.07	0.37	2.55	0.015	0.40
Safety	40	5.31 ± 1.33	4.92 ± 1.18	0.39	2.05	0.047	0.32
Intelligence	40	4.78 ± 0.87	4.58 ± 0.87	0.20	1.44	0.158	0.23
Competence	40	3.88 ± 0.90	3.75 ± 0.96	0.13	1.01	0.320	0.16
Discomfort	40	2.05 ± 1.16	2.20 ± 1.10	−0.15	−0.75	0.455	−0.12

Differences between navigation strategies were also evident across several social perception dimensions. Compared to BL navigation, SA navigation received significantly higher ratings for animacy, warmth, anthropomorphism, likeability, and perceived safety. These effects were associated with small to medium effect sizes. In contrast, no statistically significant differences were found between navigation strategies for perceived intelligence or competence. Ratings of discomfort were also comparable across conditions, with no significant increase in discomfort observed for either navigation strategy.

**FIGURE 3:**
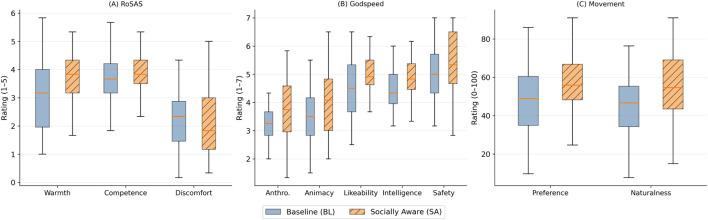
Distribution of participant ratings for Baseline (BL) and Socially Aware (SA) navigation strategies across questionnaire measures. **(A)** RoSAS dimensions, **(B)** Godspeed dimensions, and **(C)** movement-related ratings. Boxes indicate interquartile ranges, center lines denote medians, and whiskers represent data ranges. Naturalness and movement preference were rated on a 0–100 scale; RoSAS used 5-point Likert scales, and Godspeed 7-point Likert scales.

Figure 3 illustrates the distribution and variability of participant ratings across conditions and complements the statistical comparisons reported in Table 1. Taken together, these findings partly support H1a, indicating that socially aware navigation was rated higher on multiple positive social attributes and movement quality measures, including warmth, likability, naturalness, smoothness, and overall movement preference, but not perceived intelligence. H1b was not supported, as no significant differences were observed between navigation strategies in perceived comfort.

### 4.2 Exploratory analyses: participant characteristics

Exploratory analyses examined whether participant characteristics moderated perceptions across navigation conditions. Participants reported relatively low to moderate prior experience with robots, with self-reported robot knowledge averaging 2.58 out of 5 (SD = 1.08), with the majority rating their knowledge at 3 or below (n = 30 out of 40), and hands-on robot experience averaging 2.08 out of 4 (SD = 1.12). In terms of field of study, 26 participants were classified as having a technical background (e.g., computer science, robotics, engineering) and 14 as non-technical (e.g., law, psychology, medicine, business). No significant effects were found for any demographic or experience variables, including gender (all p>0.05 ), field of study (all p>0.17 ), self-reported robot knowledge (all |r|<0.27 , all p>0.09 ), or hands-on robot experience (all |r|<0.20 , all p>0.23 ). These analyses did not indicate systematic differences in the effects of navigation strategy across participant groups or levels of experience.

### 4.3 Qualitative observations from open-ended responses

Open-ended responses were analyzed to provide additional context for the quantitative findings and to examine how participants described perceived differences between navigation strategies in their own words. Across all three questions-perceived differences, preferred movement style, and suggested improvements-participants primarily referred to directly observable movement characteristics, including path selection, head orientation, speed, and proximity. Given the sample’s relatively low to moderate self-rated robotics knowledge ( M=2.58 out of 5), responses were generally expressed in experiential and interaction-related terms rather than technical descriptions of navigation or planning mechanisms.

As summarized in Table 2, responses converged around a small set of recurring movement-related themes. Many participant comments could be related to specific implemented navigation behaviors. For example, comments such as *”It felt uncomfortable when it moved behind me”*, *”I felt unsafe and a bit scared when it was behind me”*, and *”I felt much more comfortable when the robot crossed in front of me compared to behind me”* correspond closely to the approach-direction constraints implemented in the socially aware navigation strategy. Participants typically described these behaviors in terms of comfort, safety, friendliness, or social appropriateness rather than using technical navigation terminology.

**TABLE 2:** Summary of themes identified in open-ended responses across questions on perceived differences, preferred movement, and suggested improvements. Counts indicate the number of responses in which a theme was mentioned at least once per question. Example descriptions illustrate how participants articulated each theme.

Theme	Difference	Preference	Suggestion	Example description
User-oriented motion (head, gaze, acknowledgment)	20	29	18	”Movements with head rotations felt more human-like and friendly, kind of recognising the person present in the room.””For some movements, the robot did not look at me at all, and this made me feel left out.”
Movement dynamics (speed, smoothness)	9	2	9	”The turns are becoming smoother, and there are fewer clumsy movements.””Sudden stops made the robot seem less natural and more machine-like.”
Path choosing (front vs. behind, routing)	11	9	3	”I felt much, much more comfortable when the robot crossed in front of me compared to behind me.””I felt unsafe and a bit scared when it was behind me.”
Proximity and comfort	6	3	1	”Sometimes it stopped near me and looked at me.””The last time it was too near to me.”
Environmental awareness (scanning, obstacles)	9	2	2	”It stopped before changing direction and reacted when obstacles were nearby.””Some movements naturally scanned the space before reaching the barrier, resulting in smoother movement.”
Abstract technical framing (rare responses)	2	1	2	”If any RL was involved, maybe try to make a better environment setup.””It seemed to have a feedback loop which improved its sociability and smoothness.”

Similarly, participants frequently commented on user-directed motion cues such as head orientation and gaze behavior. Movements in which the robot oriented its head toward the user were described as *”more human-like and friendly”*, *”kind of recognising the person present in the room”*, or as making participants feel *”more involved.”* In contrast, the absence or timing of gaze behavior was occasionally associated with discomfort or reduced engagement, for example: *”for some movements the robot did not look at me at all and this made me feel left out”* and *”the glances sometimes seem a bit creepy.”*

Participants also frequently referred to movement dynamics using terms such as *”smooth”*, *”fluid”*, *”awkward”*, *”mechanical”*, and *”natural”*. Abrupt stops, unstable turns, or jerky motion were often described negatively. For example, participants noted that *”sudden stops made the robot seem less natural and more machine-like”* and that *”the turns are becoming smoother and there are less clumsy movements.”*

Comments explicitly referring to planning mechanisms or algorithmic processes were comparatively rare and appeared only in isolated responses from technically oriented participants, such as speculation about reinforcement learning or environment configuration. Such responses were comparatively rare and did not reflect the dominant pattern across participants.

Overall, the qualitative responses broadly supported the quantitative findings by showing that participants primarily described and evaluated the robot’s movement in social and interpersonal terms, such as comfort, friendliness, safety, or awareness. This pattern is consistent with prior research suggesting that robot navigation behavior can influence social evaluations of robots in shared environments.

## 5 Discussion

This study suggests that differences in robot navigation strategies can be perceptually meaningful to users during in-person human-robot interaction. Participants consistently differentiated between the baseline and socially aware navigation conditions across multiple social and movement-related dimensions, indicating that navigation behavior can influence how robot motion is perceived during human–robot interaction. The findings further suggest that navigation functions not only as a task-execution mechanism but also as a socially relevant aspect of robot behavior in shared environments.

The findings indicate that socially aware navigation primarily influences affective and experiential aspects of robot motion. While task-related evaluations such as perceived intelligence and competence remained stable across navigation strategies, socially informed navigation led to higher ratings on social and movement-related attributes, including warmth, likeability, animacy, perceived safety, and naturalness of motion. One plausible explanation for the absence of differences in perceived intelligence and competence is that both navigation strategies successfully achieved the task without errors or visible failures. As a result, participants may have anchored judgments of intelligence and competence to the robot’s overall task execution and platform capabilities, rather than to subtle variations in navigation behavior. This suggests that, when task performance is held constant, navigation-level adaptations may primarily shape social and experiential impressions rather than cognitive attributions related to capability Tusseyeva et al. (2024). These improvements in social perception were not accompanied by an increase in discomfort. The absence of differences in perceived comfort may, in part, be influenced by the robot platform itself. Pepper is a humanoid social robot with an expressive appearance and interaction-oriented design, which may already elicit relatively high baseline comfort levels. In this context, navigation adaptations may have had limited room to further increase comfort ratings. The role of robot embodiment in moderating how navigation behavior influences comfort warrants further investigation Nikolovska et al. (2026b).

H2 was supported by the results, with participants expressing a clear overall preference for the socially aware navigation strategy. This preference was reflected in significantly higher ratings on the movement preference item and was further echoed in the open-ended responses. Participants consistently associated preferred movements with characteristics of the socially aware strategy, including trajectories perceived as smoother and more fluid, frontal approaches, and visible acknowledgment of user presence. Importantly, smoothness was assessed only through participant perception and was not derived from an explicit trajectory smoothness metric or additional path smoothing algorithm. Both navigation strategies used the same underlying planners and trajectory generation framework, differing only in the activation of the socially informed navigation layers. These observations indicate that participants differentiated between the two navigation strategies not only at the level of individual questionnaire dimensions, but also in terms of their overall movement preference.

Beyond the partial support found for the stated hypotheses, the results suggest that participants formed evaluations of the robot’s navigation behavior based on multiple concurrently present motion cues, including interpersonal distance, approach direction, side-consistent passing behavior, and localized speed modulation. The socially aware navigation strategy evaluated in the present study combined these navigation adaptations into a single behavioral configuration rather than examining each feature independently. Consequently, the current findings do not allow conclusions regarding the individual contribution of specific navigation components. Instead, the results indicate that participants responded differently to the overall navigation styles produced by the two configurations. This highlights the value of evaluating socially aware navigation not only through isolated navigation parameters, but also through comparisons between broader navigation configurations during user interaction.

Different navigation-related behaviors appeared with varying frequency in participants’ descriptions of the robot’s motion. Comments related to approach direction, interpersonal proximity, and head orientation appeared frequently across both preference judgments and descriptions of perceived differences, while movement dynamics such as speed and smoothness were often mentioned in relation to pauses, abrupt motion changes, or perceived naturalness. These observations align with prior findings from the PRoMo questionnaire Nikolovska et al. (2025), where participants emphasized multiple socially relevant aspects of navigation behavior. However, the present study did not independently manipulate individual navigation features or quantify their relative contribution to user perception, and therefore these qualitative patterns should be interpreted descriptively rather than as evidence of differential perceptual influence.

Taken together, the findings indicate that socially aware navigation influences user perception of robot motion across multiple social and experiential dimensions. Participants consistently reported preferences and evaluations associated with observable aspects of robot motion related to proximity, path placement, and movement behavior in shared space. At the same time, the stability of perceived intelligence, competence, and comfort suggests that navigation adaptations may interact with factors such as task success and robot embodiment in shaping user judgments. Overall, the findings support the importance of considering navigation behavior as a meaningful component of human–robot interaction and motivate further investigation of socially aware navigation strategies across different robot platforms, environments, and interaction contexts.

Beyond the immediate perceptual findings, the present results may also point to the broader role of navigation behavior within human–robot interaction. In shared environments, navigation is often among the earliest and most continuously observable aspects of robot behavior, potentially shaping how users experience and evaluate a robot before any explicit interaction takes place. Variations in proximity regulation, path placement, approach direction, or movement coordination may therefore influence whether robot behavior is perceived as considerate, predictable, or socially appropriate in everyday settings. As robots become increasingly integrated into public and semi-public environments, navigation behavior may play an important role not only for physical safety and task efficiency, but also for social acceptance and user comfort during repeated encounters.

This study was conducted in a controlled indoor environment with a fixed navigation task and a single robot platform, which may limit the generalization of the findings to other contexts, tasks, or embodiments. The controlled setting enabled systematic comparison between navigation strategies and minimized confounding factors; however, shared environments in real-world applications are often more dynamic, involving moving people, changing obstacles, and less predictable interaction scenarios. Perceptual responses to robot navigation should therefore be examined in future work within more complex or crowded environments. The seated position of participants also represents a limitation of the current study. While this design was motivated by ecological validity for office-like environments, the fixed viewpoint may limit generalizability to scenarios in which users are standing or moving through the environment. Future work should examine whether the perceptual effects observed here extend to interactions with mobile users.

In addition, the study focused on short navigation encounters rather than prolonged interaction. While this design was appropriate for isolating perceptual differences based on motion alone, user evaluations may evolve over longer-term exposure as familiarity and expectations develop. Future work should examine how socially aware navigation strategies are perceived during extended interactions and in tasks involving direct collaboration.

Finally, the present work relied primarily on subjective self-report measures to assess user perception of the robot. Although these measures are well suited for capturing social and experiential judgments, combining them with behavioral or physiological measures could provide a more comprehensive picture of how navigation behavior influences comfort, trust, and engagement over time. Extending the modular navigation framework to additional social norms or cultural contexts may further clarify how user expectations shape navigation preferences in shared human–robot environments.

## 6 Conclusion

This work presented a perceptual evaluation of our socially aware navigation framework derived from empirically elicited user expectations. Our algorithm integrates multiple socially relevant navigation behaviors - including interpersonal distance regulation, approach direction constraints, side-consistent passing behavior, proximity-based speed adaptation, and user-oriented motion cues-within a single navigation configuration and evaluates how these combined behaviors influence user perception during real in-person interaction.

Compared to a baseline costmap-based obstacle avoidance strategy, the proposed socially aware navigation configuration was associated with more positive evaluations across multiple social and movement-related dimensions, including warmth, likeability, animacy, safety, naturalness of motion, and overall movement preference, while maintaining comparable ratings of competence and comfort.

The findings further suggest that navigation behavior may influence how robot motion is socially interpreted in shared environments. Participants frequently described observable motion behaviors in experiential and social terms related to comfort, attentiveness, friendliness, and awareness. At the same time, the present study did not isolate the contribution of individual navigation features, and therefore, future work is needed to better understand how specific navigation components contribute to user perception both independently and within broader navigation configurations.

Overall, the results support continued investigation of socially aware navigation strategies that combine multiple socially relevant movement behaviors within integrated navigation systems. More broadly, the findings highlight that effective robot navigation in shared environments depends not only on task completion and collision avoidance, but also on how robot motion is experienced and evaluated by nearby users. These findings may become increasingly relevant as mobile robots transition from controlled laboratory settings into everyday human-centered environments in which navigation behavior is continuously visible to nearby users. The present work further demonstrates the value of evaluating socially aware navigation using both standardized social perception measures and open-ended user feedback to better capture how navigation behavior is interpreted during real human-robot interaction.

## Data Availability

The raw data supporting the conclusions of this article will be made available by the authors, without undue reservation.
